# Foreign-Trained Dentists in the United States: Challenges and Opportunities

**DOI:** 10.3390/dj6030026

**Published:** 2018-07-01

**Authors:** Sergio Varela Kellesarian

**Affiliations:** Department of General Dentistry, Eastman Institute for Oral Health, University of Rochester, Rochester, NY 14642, USA; sergio_kellesarian@urmc.rochester.edu

**Keywords:** accreditation, dentists, diversity, education, United States

## Abstract

The aim of the present study is to review the licensing process and challenges faced by foreign-trained dentists in United States (U.S.), and how incorporating foreign-trained dentists in the dental workforce in the U.S. impacts the population’s dental care. Foreign-trained dentists must complete additional training in a Commission of Dental Accreditation recognized program offered by a U.S. dental school in order to be eligible for licensing. Foreign-trained dentists interested in seeking employment in the U.S. face numerous challenges, including stringent admission processes, high tuition costs, immigration barriers and cultural differences. Opening the U.S. dental profession to foreign-trained dentists provides several advantages, such as increasing the diversity of dentists in the U.S., expanding access to underrepresented communities, and enhancing the expertise of the profession. Foreign-trained dentists are an important resource for a U.S. government seeking to build the human capital base and make the most of global trade opportunities through a “brain gain”. Increasing the diversity in the dental profession to match the general U.S. population might improve access to dental care for minorities and poor Americans, reducing disparities in dental care.

## 1. Introduction

*“Since I recognize that graduates of foreign dental schools, US citizens or not, might make a worthwhile contribution to dentistry in this country … Still we must be careful to uphold standards of US Dentistry—the best in the world—and, therefore, should take a good look at foreign-trained dentists to be sure they measure up our standards before granting them licensure”*.

[1]

This passage is extracted from an editorial published in the Journal of the American Dental Association in the year 1977. This was the first article published in indexed literature that expressed concerns regarding the professional role of foreign-trained dentists (dentists graduating from institutions outside the United States (U.S.) and Canada) as dental/oral healthcare providers in the U.S. This editorial also emphasized the superiority of the current U.S.-based dental education system over dental institutions in many other countries [1]. Likewise, in the year 1982, Dr. Marvin Rubin, Chairman of the Council on Education of The Dental Society of the State of New York, expressed concerns regarding the licensure for foreign-trained dentists to practice clinical dentistry in the State of New York. Rubin also presented a 5-year (1975 to 1980) dramatic data, that compared the rates of failures in the clinical examination among U.S.-trained and foreign-trained dentists. The failure rate among U.S.-trained and foreign-trained dentists, ranged between 1.34% and 6.44% and 66.1% and 80.9%, respectively [2].

The concept of professional self-regulation is based on the premise that members of a profession possess specific standardized skills and competencies; which make the profession evaluate competence and performance of its members. In this regard, it is an obligation for dental health professionals to render services committed to the society [3]. The society’s trust in dental profession and the provider is essential to sustain the social contract. Maintenance of certification and revalidation are valid mechanisms to promote and maintain professional self-regulation [4,5]. In the U.S., several associations claim to represent dentists, which include the American Dental Association (ADA), American College of Dentists and the National Dental Association [6].

Licensure of foreign trained dentists created a debate in U.S. dentistry, raising concerns in globalization, diverse workforce and access to care issues. Supporters of a diverse dental workforce highlight the importance of foreign-trained dentists, valuing provider diversity and arguing increased access to care for underserved populations [7]. On the other hand, concerns have been raised in terms of patient safety and assurance of provider competence [8]. Nearly 3 decades ago, the Commission on Dental Accreditation (CODA) passed a legislation that barred foreign-trained dentists from gaining licensure (through a certification or clinical examination) to practice clinical dentistry in the U.S. Instead, foreign-trained dentists were obliged to apply and successfully complete a 2- or 3-year dental educational program in order to become licensed practitioners. These changes were introduced in order to protect the public and ensure that international dentists were adequately trained to meet U.S. standards [9,10,11]. Presently, licensing requirements for foreign-trained dentists in the U.S. are strict, confusing, complex and are conferred by individual state licensing boards. This is very different when compared with the rest of the world, where the federal governments set standards for dental education and licensure [12].

With this conflicting background, the aim of the present study is to review the licensing process and challenges faced by foreign-trained dentists in U.S. and how incorporating foreign-trained dentists in the dental workforce in the U.S. impacts the population’s dental care. It is hypothesized that reducing barriers for licensing to foreign-trained dentists can preserve population safety while improving expertise and diversification of the U.S. dental workforce together with helping to reduce healthcare disparities in the nation. 

## 2. Materials and Methods

The focused question addressed was: What are the licensing pathways and challenges faced by foreign-trained dentists in U.S. and their impact to the dental care of the U.S. population? In order to identify studies relevant to the focused question, a structured literature search without language or time restriction, up to and including May 2018, was conducted using the PubMed (National Library of Medicine), Scopus, OVID and Web of Science data-bases. All levels of available evidence, including original studies (prospective and retrospective), review articles, commentaries and letters to the editor were sought. The following Medical Subject Headings (MeSH) were used: (1) dentists, (2) education, (3) United States, (4) licensure. Other related non-MeSH terms were used to identify additional studies exploring foreign-trained dentists in U.S.: (5) foreign, (6) trained and (7) international. Boolean operators (OR, AND) were used to combine the key words mentioned above: (a) 1, AND 5 OR 7, AND 6, AND 3; (b) 1, AND 5 OR 7, AND 6, AND 2 OR 4. After the initial electronic search, the reference lists of the studies identified were hand-searched to identify further potentially relevant studies. In order to analyze the included articles, a qualitative methodological approach was used, rather than a quantitative method, due to the nature of the focused question and the studies available in the literature [13].

## 3. Results

### 3.1. Study Selection

Through the initial search, 47 potential articles were identified. After title and abstract screening, 29 publications that did not answer the focused question or were duplicates were excluded. In total 18 articles were sought, including nine articles [7,14,15,16,17,18,19,20,21] discussing workforce diversity in dentistry (Table 1); and nine articles [1,2,8,9,10,22,23,24,25] discussing licensing process for foreign-trained dentists (Table 2).

### 3.2. Pathway to Licensing

**Figure 1:**
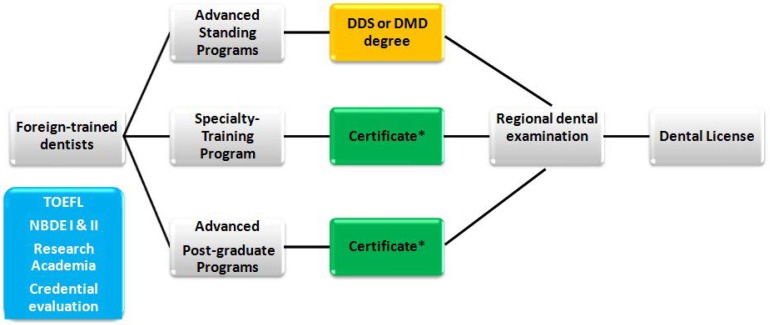
Pathways to licensing for foreign-trained dentists in the United States. * Recognized by a limited number of States.

Foreign-trained dentists must complete additional training in a CODA accredited program offered by an U.S. dental school in order to be eligible for licensing. There are three different educational pathways to licensing (Figure 1), including:

#### 3.2.1. Advanced Standing Programs

Foreign-trained dentists are allowed to gain acceptance in the second or third year of dental school, and acquire a degree in dentistry in the U.S. (Doctor of Dental Surgery (DDS) or Doctor of Medicine in Dentistry (DMD)) after completing 2 or 3 years of undergraduate study. Approximately 896 foreign nationals were admitted in 2016 in U.S. dental schools, including first-year students and advance standing programs [25].

The number of U.S. dental schools offering educational programs for foreign-trained dentists have increased substantially in the past years. According to the ADA, 32 of the 65 dental schools in U.S. offer advanced standing programs for foreign-trained dentists. Application requirements include passing scores of: (a) Test of English as a Foreign Language (TOEFL), (b) National Board Dental Examination (NBDE) Part I and Part II, and (c) transcripts from dental school evaluated by independent educational credentialing institutions. Additional requirements include psychomotor bench tests, case presentations and personal interviews [8,9,22].

#### 3.2.2. Specialty Training Programs

These allow U.S.-trained and foreign-trained dentists to combine residency post-doctoral training with research training in specific specialties of dentistry. The ADA recognizes 9 dental specialties: (a) Dental Public Health, (b) Endodontics, (c) Oral and Maxillofacial Pathology, (d) Oral and Maxillofacial Radiology, (e) Oral and Maxillofacial Surgery (OMFS), (f) Orthodontics, (g) Pediatric Dentistry, (h) Periodontology and (i) Prosthodontics. The duration of these specialty training programs may vary from 2 to 6 years depending on the program. For example, OMFS programs are a minimum of 4 years; however, 6 years OMFS programs are completed together with a Doctor of Medicine Program. For licensing of foreign-trained dentists, only a limited number of states (such as Texas and Virginia) accept successful completion of a clinical specialty program instead of an U.S. dental degree [22].

#### 3.2.3. Advanced Post-Graduate Education Programs

These include 1-year or 2-year residencies: General Practice Residency (GPR) and Advanced Education in General Dentistry (AEGD). There are also advanced programs in non-ADA-recognized specialties such as (a) Dental Anesthesiology, (b) Cosmetic Dentistry, (c) Orofacial Pain, (d) Oral Medicine, (e) Operative Dentistry, (f) Gerodontology and (g) Special Needs Dentistry [22]. Though these programs are ADA credited, they remain unendorsed for specialization. These programs usually grant a certificate of completion (not a DDS or DMD degree) which may satisfy only licensure eligibility requirements in the state where the program is located, or be recognized only by a limited number of states [9]. Several dental schools do not accept foreign-trained dentists into their programs due to state policies, and only applicants graduating from a U.S. based dental school are eligible for application [22]. Application requirements include passing scores of: TOEFL, NBDE Part I and Part II, transcripts from dental school (evaluated by independent educational credentialing institutions), grade point average, class ranking, and letters of recommendation.

Following completion of the aforementioned educational requirements, foreign-trained dentists need to complete the regional dental examination required by the State. There are 5 regional testing agencies in U.S.: (a) the Western Regional Examining Board (WREB), (b) Central Regional Dental Testing Service (CRDTS), (c) Commission of Dental Competency Assessment (CDCA) (formerly known as North East Regional Board (NERB) of Dental Examiners), (d) Southern Regional Testing Agency (SRTA), and (e) Council of Interstate Testing Agencies (CITA). Depending upon the state-defined regulations, additional requirements may be warranted, which include (a) jurisprudence exam, (b) laws and ethics exam, and (c) background check [22,23].

### 3.3. Challenges

Foreign-trained dentists interested in seeking employment in the U.S. face numerous challenges, including: stringent admission processes, high tuition costs, immigration barriers and cultural differences [22,26,27].

#### 3.3.1. Admission Process

The available openings for foreign-trained dentists in the different educational programs are limited, which leads to very competitive and stringent admission processes. Table 3 summarizes the number of applicants between 2014 and 2018 who gained admission into residency programs which participate in the Postdoctoral Dental Matching Program [26]. 

#### 3.3.2. Tuition Costs

Traditionally, U.S. public universities have 2 tiers of pricing: rates for state residents and for non-residents. However, some institutions have introduced a third, higher tier specifically for students coming from abroad. It is argued that higher tuition rates are necessary to pay for services that international students use exclusively or more intensively than others, such as the monitoring and reporting requirements to the federal government (F-1 and other non-immigrant visas, the Student and Exchange Visitor Information System (SEVIS)); and other services to a population of non-native English speakers, including the International Students and Scholar Office [27]. This same argument can be extrapolated to dentistry, where foreign trained dentists pay higher tuition than U.S. trained dentists [16]. For example, the projected tuition and fees for the 2-year international dentist program at University of Colorado for the class of 2019 is $157,260 USD ($78,630 USD each year); whereas, the yearly rates for Colorado residents and non-residents are $36,205 USD and $61,508 USD, respectively [28]. Similarly, the 2-year International Dentist Program for foreign-trained dentists in University of Buffalo requires the payment of $75,000 USD for a mandatory continuing education summer program in addition to the academic year tuition [29]. A similar scenario occurs with post-doctoral programs. For example, in Eastman Institute for Oral Health (EIOH) at University of Rochester, the annual tuition fee for a foreign-trained dentist in the 2-year AEGD program, is $15,000 USD; whereas, those residents graduated from a U.S. or Canada dental school receive an annual stipend of approximately $51,000 USD [30]. 

Furthermore, it is pertinent to mention that additional to tuition fees, travelling, relocation and living expenses in the city of the program must be also added to the total costs [9]. Moreover, scholarships, loans and financial support to foreign-trained dentists are limited [10].

#### 3.3.3. Immigration Barriers

In addition to licensure requirements, a foreign-trained dentist are required to be a permanent resident, citizen, or have a valid legal visa of the U.S. in order to practice [22]. Nonimmigrant visas (such as the F-1 student visa) are given under the condition that after gaining the special skill, the visa holder should return to their home country to serve their nation. The U.S. does not offer a direct path to permanent immigration for foreign students; sponsorship by an U.S. employer is required [10]. The H-1B visa program allows U.S. companies to employ foreign workers in different areas requiring theoretical or technical expertise. In 2017, 1169 H-1B applications were filled by dental offices. The top five companies applying for H-1B visas for dentists were: Western Dental Services, Dental Dreams, Maverick Family Dental, Jdc Healthcare, and Perfect Dental; to fill positions in only five states: California, Connecticut, Massachusetts, Pennsylvania and Texas. These five companies combined, account for 11.2% of total dental applications [31].

#### 3.3.4. Cultural Differences

Foreign-trained dentists differ from U.S. dental students in terms of cultural values, previous personal and professional life experiences, and maturity (as they are often older with families) [9,32]. These factors, including psychological and socio-cultural adjustment, may result in additional challenges to foreign trained dentists to fit in the U.S. dental school environment, affecting their academic performance [9].

### 3.4. Opportunities

Opening the U.S. dental profession to foreign-trained dentists provides several advantages. For example, foreign-trained dentists increase the diversity of dentists in the U.S., expand access to underrepresented communities, and enhance the expertise of the profession [7,19,22,33].

#### 3.4.1. Underserved Communities

The U.S. Department of Health and Human Services has designated 5000 Oral Health Professions Shortage Areas [22]. Communities with high proportions Hispanic and African American residents are 4 times more likely to have a shortage of physicians, regardless of community income [34]. It has been suggested that having more foreign-trained dentists could impact dentist practice patterns by offering increased access to rural or undeserved areas; however, there is no strong evidence to support this statement [21,25]. A recent study reported that 44% of clinically Hispanic dentists primarily treat undeserved patients at their practice [19]. Moreover, it has also been reported that minority dentists were two times more likely to accept new Medicaid patients compared with white dentists [33].

#### 3.4.2. Diversity

Disparity between the proportions of African Americans, Hispanics and American Indians in the general U.S. population and the nation’s dental profession, has been extensively reported in the literature [7,19]. These minority groups represent approximately 30% of U.S. population; however, only comprise 6% of the dental workforce [15,17]. In the year 2016, foreign-trained dentists working outside academic settings represented 5.6% of the U.S. dental workforce [25]. Studies have suggested that the presence of minority healthcare professionals is imperative to meet healthcare needs of minority communities [17,34]. However, there is not available evidence reporting that minority healthcare professionals of any origin are more likely to serve any minority community. 

#### 3.4.3. Expertise and Experience

Incorporating foreign-trained dentists might benefit U.S. training programs by increasing maturity, diversity and different perspectives on healthcare [22]. Similarly, foreign healthcare providers contribute to academics and research enhancing the U.S. medical system [16]. Approximately 13.1% of dentists working in U.S. academic settings in 2016 were identified as being foreign-trained. A significant rise compared with 2002 and 2009, where foreign-trained dentists in academia were estimated to be 3.3% and 9.1%, respectively [25].

## 4. Discussion

From the literature reviewed, it is noteworthy that foreign-trained dentists face numerous challenges to practice in the U.S. In order to become licensed, dental professionals must complete additional training in a CODA accredited program offered by a U.S. dental school. Although the number of U.S. dental schools offering educational programs for foreign trained dentists have increased substantially in the past years, the available openings are limited, which leads to a very competitive admission process. Therefore, in order to promote diversity in dental professional education and practice, effective ways to identify and address “unconscious bias” in admissions and recruitment processes are needed. It is hypothesized that increasing the number of foreign-trained dentists might help to develop awareness and ability among all dental providers to respond to patients with different values or culture. It is necessary to shift from a “cultural competence” to a real cultural proficiency and cultural humility. Diversity is essential to academic and professional excellence. A significant amount of learning occurs through informal interactions among individuals who are of different races, ethnicities, religions and backgrounds. Moreover, cultural competence cannot be effectively acquired in a relatively homogeneous environment. Dental programs must create an environment that ensures an in-depth exchange of ideas and beliefs across gender, racial ethnic, cultural and socioeconomic lines. For example, EIOH at the University of Rochester, is unique among academic health centers in the U.S. Despite the absence of an undergraduate dental school, the institution offers a variety of postdoctoral educational opportunities for U.S.-trained and foreign-trained dentists. Demographic information for EIOH residents and postdoctoral dental fellow population for the period 2017–2018 was obtained from the Registrar Office at EIOH. In total: 140 trainees, including 65 U.S.- and Canada-trained dentists, 65 foreign-trained dentists, two graduate students (Master of Science candidates) and eight preceptors. In terms of citizenship, 67 (47.8%) trainees were non-resident aliens; whereas, 73 (52.2%) trainees were U.S. Citizens or Permanent Residents; out of which 21% were from historically under-represented groups (specifically African American and Hispanic). The current trainee population represents 39 different countries (including the U.S.) and 19 different states of U.S.

Immigration and economic barriers also represent a significant limitation for foreign-trained dentists. In order to complete an educational program in U.S., international dentists need to request for an F1 or “student” visa. This visa presents strict work restrictions for the student. Moreover, students are usually non-eligible for a social security number; do not have access to scholarships and loans, and have higher tuitions compared with U.S. students/dentists. Therefore, it is necessary to develop financial aid programs to reduce the financial barriers for minority dental students and foreign trained dentists in order to increase their access to dental programs and expand the dental workforce. Furthermore, after completing the educational programs, foreign-trained dentists must be legal permanent residents, citizens, or have a valid legal U.S. visa in order to practice. It is noteworthy that there is a need of a migratory reform, shifting toward a model of “skilled-based immigration”, similar to Canada and Australia; where professionals with higher skills and competences are offered permanent residency. U.S. current policy prioritizes family reunification and set quotas for the most highly skilled immigration categories; whereas, Canada’s skill-based immigration is consistent with its continuing prospect of nation-building through human capital accumulation. This immigration process allows three-times the level of per capita immigration as the U.S. has, while at the same time maintains a stable and relatively high public tolerance to immigration. 

## 5. Conclusions

Foreign-trained dentists are an important resource for a U.S. government seeking to build the human capital base and make the most of global trade opportunities through a “brain gain”. Increasing the diversity in the dental profession to match the general U.S. population might improve access to dental care for minorities and poor Americans, reducing disparities in dental care. Further qualitative research is needed in this regard. 

## Figures and Tables

**Table 1:** Details of the reviewed articles discussing dental workforce diversity in the U.S.

Articles Discussing Dental Workforce Diversity in the U.S.			
Author	Publication Year	Journal	Aim of the Study
Casamassimo et al. [20]	2004	Journal of Dental Education	Describes the changes in pediatric dentistry faculty numbers and teaching loads between 1995 and 2002 for postdoctoral pediatric dentistry education.
Aziz [15]	2010	Journal of Oral and Maxillofacial Surgery	Reviews the racial demographic of American oral and maxillofacial surgery as it compares with the racial demographic of the U.S.
Bazargan et al. [21]	2010	BMC Health Services Research	Describes the potential impact of foreign-trained dentists have on improving access to dental care for vulnerable populations.
Al-Sowygh & Sukotjo [16]	2011	Journal of Prosthodontics	Reviews perspectives of foreign-trained dentists in comparison with U.S.-trained Dentists in Advanced Education in Prosthodontics programs on their current clinical training and future goals.
Lacy et al. [17]	2012	Journal of Dental Education	Describes an educational program implemented in Texas A & M Health Science Center Baylor College of Dentistry, to increase the number of underrepresented minorities dental students.
Ramesh et al. [18]	2014	Journal of Dental Education	Describes the transition of an oral and maxillofacial radiology course from a traditional lecture format to an interactive case-based, team-based, interdisciplinary, and intra-professional learning model in advanced dental education.
Mertz et al. [7]	2016	Journal of Public Health Dentistry	Describes sources of data on underrepresented minority dental providers in the U.S.
Garcia et al. [14]	2017	Journal of Public Health Dentistry	Reviews the underrepresented minority dentists in the U.S. workforce.
Mertz et al. [19]	2017	Journal of Public Health Dentistry	Describes the Hispanic/Latino dentist workforce in the U.S., their general practice patterns, and their contributions to oral health care for Hispanic and underserved patients.

**Table 2:** Details of the reviewed articles discussing the licensing process for foreign-trained dentists in the U.S.

Articles Discussing the Licensing Process for Foreign-Trained Dentists in the U.S.			
Author	Publication Year	Journal	Aim of the Study
Butts [1]	1977	Journal of the American Dental Association	Reviews the concerns regarding the incorporation of foreign-trained dentists as dental providers in the U.S.
Rubin [2]	1982	The New York State Dental Journal	Reviews the concerns regarding the licensure process for foreign-trained dentists to practice clinical dentistry in the State of New York.
Sweis & Guay [8]	2007	Journal of the American Dental Association	Describes the origins of foreign-trained dentists seeking licensure in the U.S.
Itaya et al. [24]	2008	Journal of Dental Education	Describes the influence of admissions criteria and cultural norms on success in an international dental studies program
Boorberg et al. [9]	2009	Journal of Dental Education	Reviews the different types of programs available to foreign-trained dentists seeking to practice in either Canada or the U.S.
Pannu et al. [10]	2013	Journal of Dental Education	Reviews the current trends in education for foreign-trained dentists in the U.S.
Allaredy et al. [22]	2014	Journal of Dental Education	Reviews the different pathways for foreign-trained dentists to pursue career in the U.S.
Catalanotto [23]	2017	Journal of Dental Education	Reviews expected changes in regulation and licensure, and its influence on future education of dentists in the U.S.
Vujicic [25]	2017	Journal of the American Dental Association	Describes future perspectives for foreign-trained dentists in the U.S.

**Table 3:** Number of students from U.S, Canadian and foreign dental schools matched to postdoctoral dental residency programs, 2014–2018. Source: National Matching Services Inc. (Toronto, ON, Canada) Postdoctoral dental matching program: statistics for applicants [26].

Year Training		Matched								Unmatched	Total
		GPR	AEGD	OMFS	PEDS	ORTHO	ANES	PERIO	PROS		
**2018**	U.S./Canada	682 (97.7%)	232 (95%)	222 (99.5%)	391 (96.7%)	256 (90.4%)	21 (100%)	93 (66.4%)	70 (60.8%)	715 (26.6%)	2682
	Non-U.S.	16 (2.2%)	12 (4.9%)	1 (0.4%)	13 (3.2%)	27 (9.5%)	0 (0%)	47 (33.5%)	45 (39.1%)	362 (69.2%)	523
**2017**	U.S./Canada	716 (97.4%)	243 (94.1%)	216 (96.8%)	384 (96.9%)	255 (90.4%)	27 (100%)	NA	NA	733 (28.4%)	2574
	Non-U.S.	19 (2.5%)	15 (5.8%)	7 (3.1%)	12 (3%)	27 (9.5%)	0 (0%)			246 (75.4%)	326
**2016**	U.S.	596 (95%)	212 (89%)	213 (96.3%)	355 (93.9%)	234 (90%)	30 (93.7%)	NA	NA	645 (28.2%)	2285
	Non-U.S.	31 (4.9%)	26 (10.9%)	8 (3.6%)	23 (6%)	26 (10%)	2 (6.2%)			221 (65.5%)	337
**2015**	U.S.	591 (95.0%)	182 (79.8%)	209 (97.2%)	359 (93.4%)	237 (88.4%)	22 (88%)	NA	NA	639 (28.5%)	2239
	Non-U.S.	31 (4.9%)	46 (20.1%)	6 (2.7%)	25 (6.5%)	31 (11.5%)	3 (12%)			283 (66.5%)	425
**2014**	U.S.	619 (95.6%)	164 (84.1%)	216 (96.8%)	357 (95.9%)	218 (86.1%)	27 (87%)	NA	NA	693 (30.2%)	2294
	Non-U.S.	28 (4.3%)	31 (15.8%)	7 (3.1%)	15 (4%)	35 (13.8%)	4 (12.9%)			285 (70.3%)	405

GPR: General Practice Residency; AEGD: Advanced Education in General Dentistry; OMFS: Oral and Maxillofacial Surgery; PEDS: Pediatric Dentistry; ORTHO: Orthodontics; ANES: Anesthesiology; PERIO: Periodontics; PROS: Prosthodontics; NA: not available.
